# Construction and analysis of dynamic transcription factor regulatory networks in the progression of glioma

**DOI:** 10.1038/srep15953

**Published:** 2015-11-03

**Authors:** Yongsheng Li, Tingting Shao, Chunjie Jiang, Jing Bai, Zishan Wang, Jinwen Zhang, Lili Zhang, Zheng Zhao, Juan Xu, Xia Li

**Affiliations:** 1 College of Bioinformatics Science and Technology and Bio-Pharmaceutical Key Laboratory of Heilongjiang Province, Harbin Medical University.

## Abstract

The combinatorial cross-regulation of transcription factors (TFs) plays an important role in cellular identity and function; however, the dynamic regulation of TFs during glioma progression remains largely unknown. Here, we used the genome-wide expression of TFs to construct an extensive human TF network comprising interactions among 513 TFs and to analyse the dynamics of the TF-TF network during glioma progression. We found that the TF regulatory networks share a common architecture and that the topological structures are conserved. Strikingly, despite the conservation of the network architecture, TF regulatory networks are highly grade specific, and TF circuitry motifs are dynamically rewired during glioma progression. In addition, the most frequently observed structure in the grade-specific TF networks was the feedforward loop (FFL). We described applications that show how investigating the behaviour of FFLs in glioblastoma can reveal FFLs (such as RARG-NR1I2-CDX2) that are associated with prognosis. We constructed comprehensive TF-TF networks and systematically analysed the circuitry, dynamics, and topological principles of the networks during glioma progression, which will further enhance our understanding of the functions of TFs in glioma.

Glioma is the most common and fatal primary brain tumour; approximately 10,000 new cases of high-grade or malignant glioma occur each year^1^,^2^,^3^. Significant new evidence suggests that the dysregulation of gene expression palys critical roles in the development of glioma^4^. Sequence-specific transcription factor (TF) binding performs key functions in gene expression control. In particular, a TF can interact with and regulate another TF, and such cross-regulation among TFs defines the regulatory subnetworks that underlie complex diseases^5^,^6^,^7^,^8^. However, despite their central roles in cellular identity and function, both the structure of human TF-TF networks and their critical functions in the progression of glioma are largely undefined.

Gene expression-based studies have provided profiles of thousands of individual transcripts, thus revealing novel genes that are dysregulated, concordant with other clinical features of gliomas, such as histological grade and patient survival^9^,^10^,^11^. In particular, TFs play important roles in the transcriptional networks that regulate gene expression and modify and control cancer phenotypes. Differentially expressed TFs in glioma and their downstream gene targets may be potential therapeutic biomarkers of glioma. For example, TP53, SP1, JUN, STAT3, and SPI1 were identified as crucial TFs involved in the development of glioma^12^. In addition, some TFs were found to play key roles in specific grades of glioma. The TF YY1 has previously been identified as a regulator of the proneural-specific expression pattern in glioma^13^. This TF plays an essential role in oligodendrocyte progenitor differentiation. Moreover, E2F1 and E2F3 are both master regulators and promote cell-cycle progression, and they were amplified in transgenic mouse models of glioma^14^,^15^. These candidate TFs and their downstream target genes may play important roles in the progression of glioma and could be potential biomarkers for clinical treatment.

The acquisition of more genome-wide expression datasets from glioma patients, has demonstrated that the processes of tumorigenesis and progression likely involve the coordinated dysregulation of molecular networks^16^,^17^ rather than single genes. Therefore, the abnormal expression of single genes may be better viewed collectively at the molecualr network level^18^. In addition, in contrast to single gene/protein targets for the treatment of glioma, these networks establish broader, multi-target pathways for glioma therapy^19^. In contrast to the entire transcription regulatory network, the subnetworks are formed by interactions among the TFs that are expressed in a given cell type, and these subnetworks make the cell system nimble and robust^20^. Although many transcriptional regulations are ignored in simple analyses of TF-TF regulation subnetworks, research has demonstrated that the cross-regulation among TFs defines a core subnetwork that plays important roles in cellular identity and complex functions, such as development, differentiation and complex diseases. Moreover, Shane *et al.* demonstrated that human TF networks are highly cell specific and are driven by cohorts of TFs, including TFs with previously unrecognized roles in the control of cellular identity^5^. Although regulatory networks provide a representation of molecular interactions, they appear to undergo dynamic reconfiguration in specific contexts^21^. Through an analysis of the transcriptional network for the mesenchymal transformation of brain tumours, Califano *et al.* identified the transcriptional module that controls the expression of the mesenchymal signature^22^. In addition, the transcriptional regulatory network of proneural glioma determines which genetic alterations are selected during tumour progression^4^. These results indicate that constructing and anlysing of the glioma-progression-associated TF-TF regulatory network might be necessary to understand the mechanism underlying glioma progression. Unfortunately, studying the factors that influence the malignant progression of glioma is limited by the poor reporting of relevant data regarding the expression of genes that are simultaneously profiled across samples with different grades.

To clarify this issue, we reanalysed the extensive dataset obtained through the mRNA profiling of a 160-patient cohort with different grades of glioma in China that was generated for one of our recent studies^5^. This data and the TF regulation information were integrated, and we constructed TF-TF regulatory networks for each grade. By analysing the grade-specific TF regulatory networks, we found that all three of these TF-TF networks share conserved network structures and a common architecture. However, the constructed TF-TF networks are highly grade selective, and the network motifs dynamically rewire during glioma progression. We then found an association between some network motifs, especially feedforward loops (FFLs), and patient survival. These systematic analyses provide novel insight into regulatory mechanisms at the TF level during glioma progression, and both the method and predictions that were generated in the current study could serve as important resources for future experimental dissection of TF functions during glioma progression.

## Results

### Comprehensive mapping of TF networks during glioma progression

**Figure 1:**
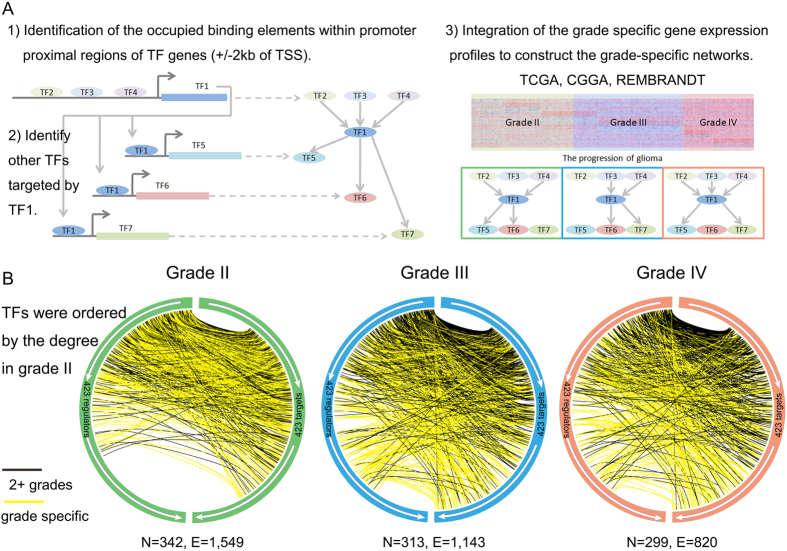
Construction of comprehensive transcriptional regulatory networks during glioma progression. (**A**) Schematic for the construction of TF regulatory networks during glioma progression. The network nodes represent TFs. Each TF can regulate other TFs and can also be regulated by other TFs; these cross-regulations formed regulatory networks. The sequence-binding information and the genome-wide gene expression were integrated. (**B**) Grade-specific versus shared regulatory interactions in the TF networks of the three glioma grades. The solid yellow lines represent TF-TF connections that were grade specific, and the black lines represent TF connections that occurred in more than two grades. The order of TFs along each circular axis is sorted by their degree in grade II.

To evaluate the dynamic rewiring of TF circuits during the progression of glioma, we integrated genome-wide gene expression profiles with sequence binding information (Fig. 1A). We used the annotated database (TRANSFAC) of TF-binding motifs to infer TF-TF regulations through Match^23^. Consequently, we obtained 88,971 regulations among 522 TFs. However, evidence of physical binding does not directly imply transcriptional functional activity of the TFs. Exploring the regulatory relationships among TFs that are specifically co-expressed in the same glioma grade could offer useful information to elucidate the role of TF-TF regulation in glioma progression. Through an analysis of the TF-TF coexpression in each grade, we observed distinct distributions of correlation coefficients for the three grades (Fig. S1). Moreover, the distributions were distinct for different datasets, implying the genetic heterogeneity of different patients.

Similar to previous studies, we used |Pearson’s coefficient|>0.5 and p < 0.01 to define a link between co-expressed TFs in the regulatory network. To obtain a robust regulatory network of different grades, a regulatory interaction between TFs was only established if correlation criteria were met in two independent datasets. By incorporating physical binding information, we obtained grade-specific TF regulatory networks (Fig. 1B and Fig. S2). Although 88,971 interactions were identified based on sequence binding, only a fraction (0.92%–1.74%) of these potential edges were activated in each grade. Specifically, 1,549 regulations among 342 TFs, 1,143 regulations among 313 TFs and 820 regulations among 299 TFs were obtained for grades II, III, and IV, respectively.

### Common topological architecture of grade-specific TF-TF regulatory networks

**Figure 2:**
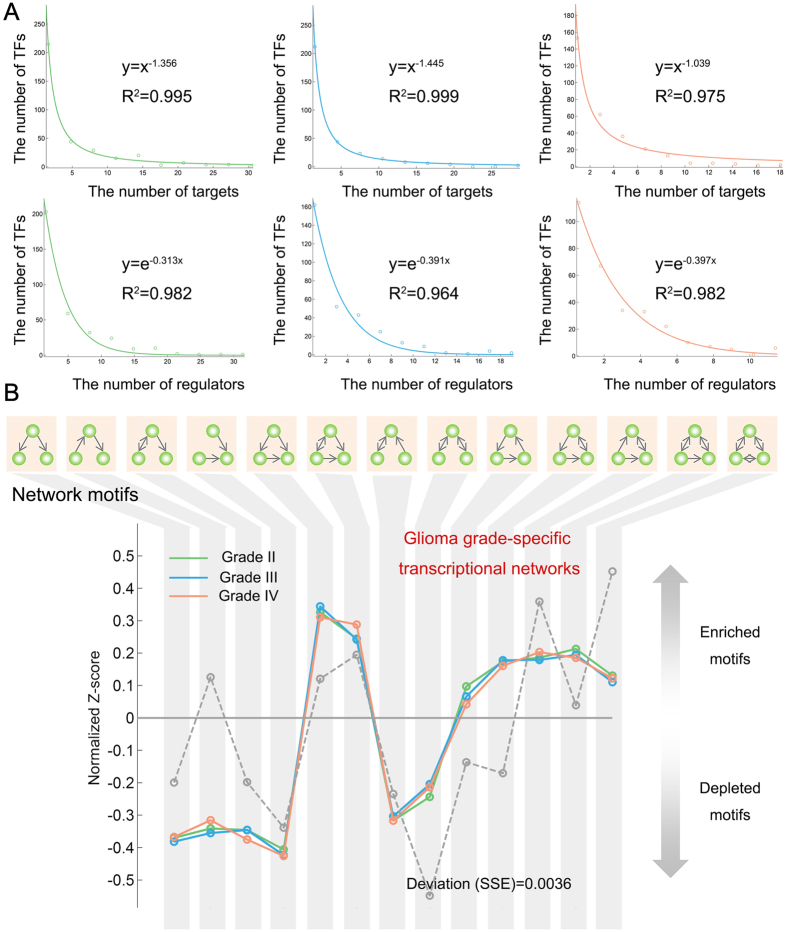
The TF regulatory networks show conserved structures and motif composition. (**A**) The in-degree and out-degree distributions of TFs in three regulatory networks. (**B**) Network motif enrichment. The relative enrichment or depletion of the 13 possible three-node motifs within the TF-TF regulatory network for each grade is shown. The dashed line represents the distribution of the Z-scores of the sequence-binding network of TFs.

An analysis of the topological features of the TF regulatory networks across each grade revealed some common topological features of the TF-TF network. We found that 48.83%–56.73% of the TFs regulate at least two targets, and approximately 61.87%–64.91% of TFs are co-regulated by 2 or more other TFs. Most TFs are connected and form a large connecting subnetwork. These results indicate a complicated combination in terms of both target multiplicity and TF cooperation. We evaluated the out-degree distribution of TF regulators and the in-degree distribution of TF targets and observed a power law and an exponential distribution, respectively. These results indicate that most TFs have few targets and somewhat more regulators. Moreover, the out-degree and in-degree distributions were conserved in the three grades (Fig. 2A). Therefore, similar to many large-scale networks, the grade-specific TF regulatory networks display scale-free characteristics; this result indicates that these networks are not random but are characterized by a core set of structural organizing principles that distinguish them from randomly linked networks.

Complex regulatory networks from diverse organisms are mainly formed by a set of simple building blocks termed network motifs^24^. Network motifs are simple regulatory subnetworks, and the topology of a given regulatory network can be reflected by the distribution of the normalized frequencies of network motifs. We therefore analysed the topology of the grade-specific TF regulatory networks. We first extracted the 13 possible three-node motifs and then computed the relative frequency and relative enrichment or depletion of each motif within each grade-specific network. Then, we compared the results of the network for each grade and found that the topologies of the TF networks in the three grades were strikingly similar (Fig. 2B). However, when we recomputed these values solely from the TF binding sites within the promoter regions of each TF without considering the grade-specific coexpression, remarkable similarity of the motif distribution was almost lost (Fig. 2B). This result affirms the criticality of using the grade-specific expression to obtain biologically meaningful conclusions from networks. These results highlight the observation that grade-specific TF-TF networks are highly similar in their structures and organizing principles.

### Dynamic rewiring of transcription regulatory networks during glioma progression

**Figure 3:**
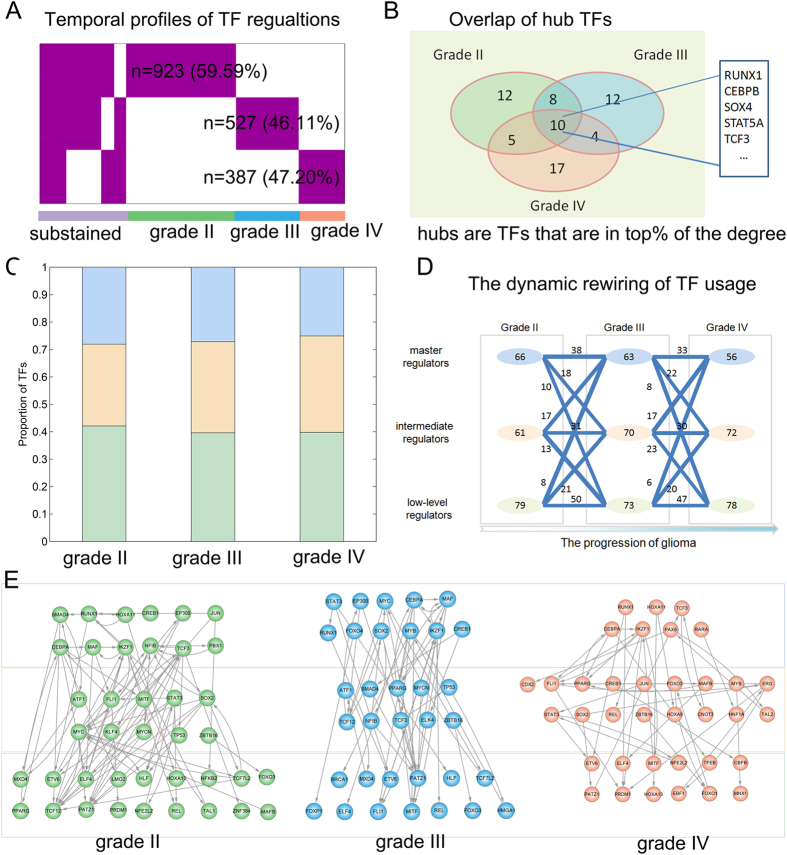
Dynamic network rewiring of TFs during glioma progression. (**A**) Network snapshots. Each column corresponds to a TF-TF interaction that occurs in at least one grade network; rows correspond to glioma grade. A purple entry indicates active interactions in the network. (**B**) The hub TFs in each grade-specific network were defined as those in which the degrees were in the top 10%. This Venn diagram shows the overlap of hub TFs for the three grades. (**C**) The proportions of TFs in each layer. Blue, master regulators; orange, intermediate regulators and green, low-level regulators. (**D**) The rewiring of regulators during glioma progression. (**E**) The hierarchical structure of cancer-associated TF regulatory networks during glioma progression. The TFs are assigned by their layer in each grade.

Although grade-specific networks share common topological features, we found that most TF-TF interactions are unique to a specific grade (Fig. 3A and Fig. S3). Approximately 26.78% of the edges were common to more than two glioma grades. In addition, we found substantial difference in the degree of TFs between different glioma grades (Fig. 1B). Because network hub nodes have been found to play important roles in many networks, we identified hub TFs in each grade-specific network. Generally, these TF hubs manifest grade-specific behaviours (Fig. 3B). Only ten hubs (such as RUNX1, CEBPB and SOX4) were shared by these three grades. In addition, the TF-TF regulations that are unique to grades typically formed a highly connected subnetwork (Fig. S4), implying that grade-specific regulatory differences are not caused by the actions of a few TFs but rather by TF-TF interactions.

Because the regulome is directed by networks, it is of particular interest to examine whether the transcription regulatory networks in the progression of glioma harbour a hierarchical structure and if so, how that hierarchy is organized^25^,^26^. In addition, TFs in different hierarchical levels are with different roles. For example, Jothi *et al.* demonstrated that TFs at the top-level in yeast are more likely to be essential and are more conserved^27^. Next, we used simulated annealing to reveal the organization of TFs in three layers of master-regulators, intermediate regulators, and low-level regulators^28^ (details in methods). The algorithm found a similar proportion of three types of regulators in three grades (Fig. 3C), with approximately 40% of TFs at the top layer of the network in each grade. However, the composition of TFs at each level was dynamically rewired in the progression of glioma (Fig. 3D). In total, 206 TFs were observed in all three grades. Of these TFs in each level, only approximately 30% were retained in the progression of glioma, indicating that the TFs in each level are highly grade specific, even between adjacent grades. The dynamic grade-specific TF usage in each level suggested that the transcription regulatory network changes rapidly between grades. In addition, the TF overlaps in each level between distant grades were lower than those of adjacent grades (Fig. S5), suggesting that the rewiring of TF levels in adjacent grades occurs more frequently than in distant grades.

Next, we focused on the regulatory subnetwork of known cancer TFs, and we found dynamic rewiring of the transcription regulatory subnetwork of these TFs during glioma progression. The position of some TFs in the subnetwork changed with glioma progression. For instance, STAT3 has emerged as a key initiator and master regulator of the mesenchymal transformation in malignant gliomas^22^. We observed that STAT3 was located in the intermediate level in grade II, and changed to the top level in grade III (Fig. 3E). This location change may contribute to the progression because the changes corresponds to activation of the STAT3 pathway. Another example is TF-CREB1, which underwent a top-to-intermediate transition during glioma progression. CREB1 plays key roles in glioma^29^, and CREB1 is essential for glioma cell growth and cell survival *in vitro* and is critical for gliomagenesis *in vivo*^30^. The grade specificity and rapid level transitions of TFs suggest that dynamic rewiring of the regulatory network is an important mechanism for glioma progression.

### Dynamic composition of FFLs during glioma progression

**Figure 4:**
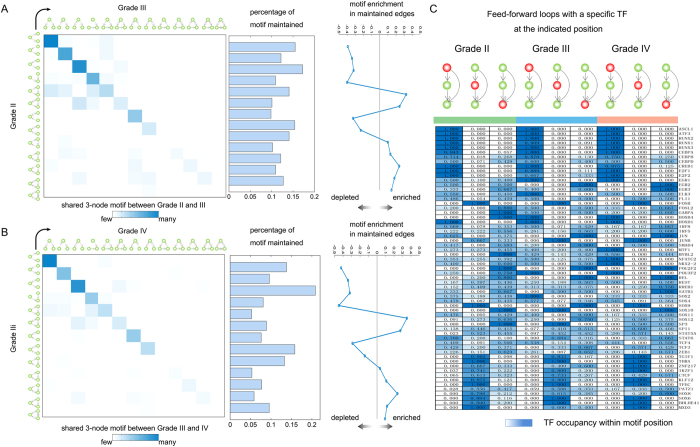
Dynamic network motif rewiring during glioma progression. (**A**) Left: frequency with which individual three-node motifs are identically maintained between grades II and III. Middle: percentage of specific three-node motifs that were identically maintained between grades II and III. Right: enrichment of the three-node motif in a network that was constructed using edges that were present in grades II and III. (**B**) Simialr to A, but for grades III and IV. (**C**) Frequency with which TFs from FFLs occupy different positions (driver, first passenger, and second passenger) for each glioma grade.

Next, to assess whether the conserved network topological architecture (Fig. 2) in each grade was driven by a common core of regulatory interactions, we identified all instances of each three-node network motif within each grade, extracted the constituent TFs and computed how the same TFs were connected for each grade. This comparison revealed that despite the conservation of the overall network architecture among the three grades, specific combinations of TFs comprising individual regulatory motifs underwent substantial remodelling during glioma progression (Fig. 4A,B); this remodeling was greater than that observed among individual edges. Overall, approximately 15% of the combinations of three TFs that were found in either grades II and III or grades III and IV were maintained. For example, FFLs are one of the most important network motifs in biological regulatory networks; biological information is propagated forward from the top TF through the middle TF to the bottom TF, with direct top TF-to-bottom TF reinforcement^31^. Indeed, only approximately 2.82% of all observed FFLs across the three grades were common (Fig. S6). We next explored the enrichment of three-node circuits in a network that was constructed using edges that were present in two grades (II and III; III and IV). The TF circuits that were conserved between grades were enriched in seven major network motif types, including FFLs, the ‘regulated mutual’ motif and the ‘clique’ motif.

In addition, we found that 61 common TFs formed the FFLs in the three studied grades of glioma. However, these TFs also demonstrated grade-specific behaviours. Rather than simply measuring the degree of these TFs, we sought to measure the behaviour of TFs within each glioma grade by determining their topological position within FFLs. For each grade, we computed the relative frequency of FFLs containing a specific TF at each of the three FFL positions (top, middle or bottom). We found that although the positions of some TFs were preseved, some TFs, including some cancer-related TFs, performed different roles during glioma progression (Fig. 4C). For example, ATF3, RUNX1 and CEBPA were likely to locate in the top position of the FFLs in all three grades, and RUNX1 preserved high connectivity in all regulatory networks, indicating that this TF may play critical roles in glioma^32^. Moreover, we observed that some TFs showed dynamic transformation during the glioma progression. For example, HOXB4 was frequently located on the bottom of the FFL in grade II, but tended to locate in the top position in grade III and grade IV. Another example is TF-POU3F2 (Fig. 4C), whose encoded protein is involved in neuronal differentiation and enhances the activation of corticotrophin-releasing-hormone regulated genes. Taken together, these observations highlight the importance of analysing grade-specific regulatory networks to explore the mechanism of glioma progression.

### TF FFL biomarkers associated with the survival of glioblastoma (GBM)

**Figure 5:**
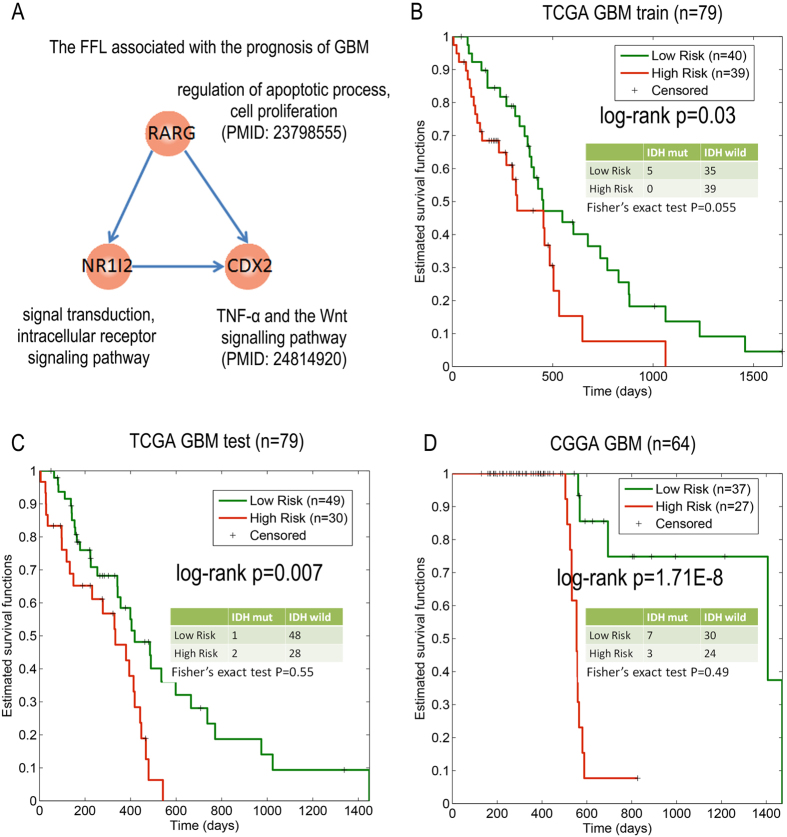
Network motifs are associated with the survival of grade-IV patients. (**A**) The FFL motif (RARG-NR1I2-CDX2) and its functions. (**B**) Kaplan-Meier survival plots were generated for this motif using the TCGA training dataset. (**C**) Kaplan-Meier survival plots were generated for this motif using the TCGA test dataset. (**D**) Kaplan-Meier survival plots were generated for this motif using the CGGA dataset.

We found that the most frequently observed structure in the grade-specific TF networks was the FFL structure. This structure has several important functions in a biological network: detecting persistent signals, filtering noise signals and accelerating response. FFLs play an essential role in the central nervous system. Thus, we next explored whether some FFL motifs were associated with the survival of grade IV-GBM. The grade-IV specific network contains 117 FFLs. We found that the FFL (RARG-NR1I2-CDX2) motif could distinguish the GBM patients with different survival times at a significance level of 0.05 in the training and testing datasets of the TCGA dataset (Fig. 5A,B). The survival-prediction capability of this FFL was validated in the test and CGGA datasets (Fig. 5B,C). In addition, when we used the CGGA dataset to obtain training and test datasets, and using the TCGA dataset for independent validation, we also identified this FFL (Fig. S7). RARG plays an important role in the proliferation, metastasis, and chemoresistance of cholangiocarcinoma through the simultaneous activation of the Akt/NF-kappaB and Wnt/beta-catenin pathways^33^. Moreover, the TFs in the FFL could not help to differentiate patients with good prognosis from those with poor prognosis. In the RARG-NR1I2-CDX2 motif, the Cox p-values for these three TFs were 0.84, 0.06 and 0.176 for the TCGA training dataset. We found that the log-rank p-values for this motif in the training and testing of the TCGA dataset were 0.03 and 0.007, respectively. These results suggest that when using a biological network as the searching platform, network-based biomarkers (i.e., FFLs) are expected to be more effective than individual TFs. This result is consistent with previous studies in which network-based biomarkers were more robust and provided valuable insights into the molecular mechanisms of disease^34^,^35^.

Previous studies have shown that patients with IDH mutations have much better survival rates and represent a distinct glioma grade compared with those for wild-type IDH tumours. Next, we determined whether the survival association of this FFL with survival depends on the IDH mutation status; the connection between the prognosis associated FFL and IDH mutation status was assessed by Fisher’s exact test. Here, we found that no distinct difference in the IDH mutation states in the low- and high- risk groups in the training (p = 0.055), test (p = 0.55) and total CGGA datasets (p = 0.49). These results suggest that this FFL may play key roles in glioma progression, and may be a potential target for glioma therapy.

## Discussion

TF-TF regulations form basic regulatory networks for biological systems, and the dysregulation of TF-TF regulatory networks plays critical roles in various types of cancer. However, despite their critical role in human complex diseases, human TF regulatory networks during glioma progression are presently poorly understood. An enduring frustration is the lack of the genome-wide TF expression data for patients with different glioma grades. Here, we reanalyzed the TF expression profiles in 160 Chinese glioma patients generated by one of our recent studies. Integrating this information with the sequence binding information and other two independent gene expression datasets from TCGA and REMBRANDT, we constructed the grade-specific TF regulatory networks for glioma grade II, III and IV. The networks that we constructed here for the three grades provide an extensive resource for analysing the circuitry, dynamics, and topological principles of the human TF regulatory network during glioma progression.

Our comparative analysis of transcriptional regulatory networks has revealed several key features of human TF regulation in glioma. First, we found that human transcription regulatory networks of glioma are markedly grade specific, with 47.20%–59.59% of all regulatory interactions only being observed in specific grades. This finding highlights the regulatory diversity during glioma progression and the importance of analysing grade-specific TF-TF regulatory networks. In contrast to the high regulatory diversity between different grades of glioma, we found that the regulatory networks of all grades share a common network topological architecture. In addition, the network motif patterns were conserved during the glioma progression. To explore whether some regulatory interactions might drive the conserved topological architecture during glioma progression, we compared network motifs between adjacent grades. These findings suggest that a distinct set of the network motifs for each grade independently specifies the conserved topological architecture of the TF-TF networks.

Moreover, we found that FFLs were the most observed network motifs in each grade. Through an analysis of the FFLs in GBM, we identified the prognosis-associated FFLs. Some FFLs were independently validated using an independent dataset, providing new potential targets for glioma therapy.

In addition, although grade-IV gliomas are mostly associated with wild-type, and grade II- and III are associated with mutated IDH, all of the grades include both cancer types. The glioma grades for cases wild-type are distinct from those of cases with mutated IDH, and we further reconstructed both grade-specific and IDH wild-type or IDH mutated networks based on the CGGA dataset. As a result, similar to the TF regulatory network in the main text, the IDH wild-type and IDH mutated grade-specific networks had a relatively low overlap among the three grades (Fig. S8). Grade-II-specific regulations predominanted in IDH mutated samples, whereas grade-IV regulations predominanted in wild-type IDH samples (Table S1). Through further analyses of these regulatory networks, we observed that all six regulatory networks also shared the common network topological architecture of the networks in the main text. In addition, the network motif patterns were conserved during glioma progression. These findings reveal the regulatory diversity during glioma progression and highlight the importance of considering IDH information when analysing grade-specific TF-TF regulatory networks.

Despite the successes of our approach, some additional steps could be used to improve our understanding of the TF regulation during glioma progression. As the number of the datasets of ChIP-Seq^36^ and DNaseI footprint maps increases, the regulatory networks derived from these datasets may be added to map and analyse grade-specific regulatory networks in cancer. The approach that we utilized in this study does not consider indirect regulatory interactions among TFs that may also affect the expression of a given TF. The identification of such indirect interactions among TFs may provide more details for modelling such interactions. In summary, our current results provided the grade-specific TF networks and a resource to analyse the circuitry, dynamics, and topological principles of the human TF regulatory network during glioma progression. Systematic analyses provided novel insight into regulatory mechanisms during glioma progression, and both the method and networks that are generated in the current study have the potential to provide novel insight into human TF regulation during glioma progression.

## Materials and Methods

### Genome-wide gene expression during glioma progression

The genome-wide gene expression profiles of 160 glioma patients were obtained from one of our previous studies^18^. Briefly, all of these patients were from Beijing Tiantan Hospital. The samples were histologically graded according to the WHO classification of tumours of the nervous systems and included 63 grade-II patients, 33 grade-III patients and 64 GBM patients. Written informed consent was obtained from all donors. Clinical investigations were conducted after approval by the local research ethics committee (Harbin Medical University) and were in accordance with ethical principles. Genome-wide mRNA expression profiles were obtained using an Agilent Whole Human Genome Array. The probes were filtered to remove non-specific probes and probes lacking association with Entrez Gene IDs. In addition, if multiple probes corresponded to a single gene, the expression values of these redundant probes were averaged. The expression values for each gene were background subtracted, quantile-normalized and log-transformed. Additionally, the top 1% low expressed genes were excluded. In total, 515 TFs were expressed in the array. All of the data were deposited in the Chinese Glioma Genome Atlas (CGGA, http://www.cgga.org.cn/), which is a publicly available database that focuses on glioma in China. The sample IDs, IDH mutation status and clinical information of the samples that were used in this study are provided in Table S2.

In addition, two additional sets of gene expression data of different grades of glioma patients were obtained from The Cancer Genome Atlas (TCGA) and The REpository of Molecular BRAin Neoplasia DaTa (REMBRANDT) projects. For the TCGA dataset, whole-genome gene expression profiles were obtained for 78 grade-II, 94 grade-III and 158 grade-IV glioma patients using RNA-seq V2. The level-3 datasets were directly downloaded and then log-transformed. For the REMBRANDT dataset, we obtained the expression as measured by Array Platforms (HG-U133_Plus_2) for 90 grade-II, 68 grade-III and 125 grade-IV patients. Gene expression analysis was performed using packages in Bioconductor^37^. The Robust Multichip Averaging algorithm (RMA) with quantile normalization was used for the normalization of the Affymetrix microarrays^38^. Additionally, the top 1% low-expressed genes in these two datasets were also excluded.

### Construction of the TF regulatory network

To construct the TF regulatory network for each grade, we first obtained 523 TFs with known motif binding information from TRANSFAC^39^. We then downloaded the defined promoter region (−2000/+2000bp around the transcription start site, TSS) of these TFs from UCSC Table Browser^40^. Next, we performed a binding site search using the Match^TM^ software that is integrated into TRANSFAC Professional. For the purpose of this study, we used a pre-calculated cut-off to minimize false positive matches (minFP) and create a high-quality matrix as described in previous studies^41^. To restrict the search, we also required a core score of 1.00 and a matrix score of 0.95.

To identify the functional regulations among TFs, we combined both the computational target predictions at the sequence level and the expression relationships between regulators and targets in the context of glioma progression (Fig. 1A). Thus, the Spearman correlation coefficients were calculated for each predicted TF-TF pair in each dataset, and the threshold was set to >0.5 or <−0.5 with a corresponding p-value of <0.01. In our study, regulatory interaction between the TFs was established only if the correlation criteria were met in two independent datasets. Finally, after assembling all of the functional TF-TF pairs for each grade, we generated the TF regulatory networks for three grades of glioma. These networks are directed graphs. A directed edge (connection) from a regulatory TF to one of its TF targets exists if their relationship is functionally regulated. To facilitate the analysis of the topological structure of the networks, the weights of all of the edges were set to 1.

### Visualization and topological features of the TF regulatory network

TF regulatory network of each grade was visualized using Circos^42^. Circular plots were formed by two parts, and each half was divided into 423 points, with one point representing a TF. Solid lines linking the left and right parts represent TF-TF interactions. The solid yellow lines are TF-TF connections that were specific to the indicated glioma grade. The solid black lines represent TF-TF connections that occurred in more than two grades. The TFs are ordered by their degree in the grade-II network. The in-degree of a TF is defined as the number of regulators that regulated the TF, and the out-degree is defined the number of genes that it regulated.

### Significance profiles of three-node motifs

The self-interactions of TFs were removed from each grade-specific network, and the mfinder software tool was used for network motif analysis^24^. We focused on the 13 possible network motifs with three TFs (three-node network motifs) in our current study. We calculated a z-score for each network motif, and 1000 randomized networks of the same size were used to estimate a null distribution. Then, the z-score was vectorized for every grade and normalized each to unit length to create triad significance profiles as described by Milo *et al.*^24^. Similar to the study published by Neph *et al.*^5^, if the motif type had a normalized Z-score above 0, it was considered as enriched. Otherwise, if the motif type had a normalized Z-score below 0, it was considered depleted.

### Classification of TF regulators based on network topology

All of the TFs in each grade-specifc network were classified into three levels based on their network topology. This process was performed using the method proposed by Snyder *et al.*^28^. Briefly, we computed the out-degree (O) and in-degree (I) of each TF and further defined a normalized ‘hierarchy height’ metric, h = (O − I)/(O + I). The ‘master regulators’ are those TFs that regulate many other TFs (h~1), ‘low-level regulators’ are more regulated than regulating (h ~ −1), and ‘intermediate-level regulators’ are both regulated and regulating (h ~ 0). The code that was used for the analysis was downloaded from http://encodenets.gersteinlab.org/enets16.hierarchy_levels.m.

### Identification of prognosis associated FFL

To identify the prognosis-associated FFL signature in grade IV-GBM, the specimens in the TCGA dataset were randomly assigned to a training data set or a test dataset. The training and test subsets were required to have approximately the same size (Table S3). According to previous studies^43^,^44^, we used the splitting strategy instead of cross-validation because there was no overlap between the training and test sample sets; this lack of overlap was an advantage over cross-validation.

Next, we aimed to identify the FFL signature from the training sets and tested them using the testing datasets. All of the FFLs that were identified in the GBM TF regulatory network were considered candidate signatures. For each FFL, we first used univariate Cox regression analysis to evaluate the association between survival and the expression level of each TF in FFL. Then, a mathematical formula for survival prediction was constructed that considered both the strength and positive or negative association of each TF with survival. The regulatory relationships between the three nodes in FFL motifs were not further considered. Specifically, we assigned a risk score to each patient according to a linear combination of TF expression values as weighted by regression coefficients. The risk score for each patient was calculated as follows:


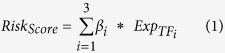


where 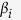
is the Cox regression coefficient of TF *i* in the training set, and 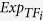 is the expression of TF *i* in the patient. All of the patients in the training data set were thus dichotomized into high-risk and low-risk groups using the median risk score as the threshold. Then, the coefficient and threshold values that were derived from the training sets were directly applied to the TF expression data of the testing sets to divide the patients in the testing sets into high-risk and low-risk groups. The Kaplan-Meier method was used to estimate the overall survival time for the two groups. The difference in the survival times was compared with the log-rank test. FFLs with p-values less than 0.05 in the training and test datasets were identified as prognostic biomarkers.

### Validation of the TF FFL prognostic biomarkers in independent datasets

To validate the TF FFL prognostic biomarkers in independent datasets, we first split the TCGA dataset into training and test datasets to identify the FFL biomarkers and then validated these biomarkers in the CGGA dataset. The expression of TFs in the FFL prognostic biomarkers was extracted. We then clustered the gene expression profiles by the k-mean method (k = 2) in the validation dataset. The patients were divided into two groups, and the difference in the survival times was compared with the log-rank test. The FFLs with p-values of less than 0.05 were analysed futher.

In addition, the CGGA dataset was also split into training and test datasets to identify the FFL biomarkers, which were then validated using the TCGA datasets. The two subsets of FFL biomarkers were overlapped to obtain the final ones.

## Additional Information

**How to cite this article**: Li, Y. *et al.* Construction and analysis of dynamic transcription factor regulatory networks in the progression of glioma. *Sci. Rep.* **5**, 15953; doi: 10.1038/srep15953 (2015).

## Supplementary Material

Supplementary Information

Supplementary Table S2

## References

[b1] Cancer Genome Atlas Research N. Comprehensive genomic characterization defines human glioblastoma genes and core pathways. Nature 455, 1061–8 (2008). 18772890 10.1038/nature07385 PMC2671642

[b2] Parsons D. W. *et al.* An integrated genomic analysis of human glioblastoma multiforme. Science 321, 1807–12 (2008). 18772396 10.1126/science.1164382 PMC2820389

[b3] Zong H., Verhaak R. G. & Canoll P. The cellular origin for malignant glioma and prospects for clinical advancements. Expert Rev Mol Diagn 12, 383–94 (2012). 22616703 10.1586/erm.12.30 PMC3368274

[b4] Sonabend A. M. *et al.* The transcriptional regulatory network of proneural glioma determines the genetic alterations selected during tumor progression. Cancer Res 74, 1440–51 (2014). 24390738 10.1158/0008-5472.CAN-13-2150 PMC3981545

[b5] Neph S. *et al.* Circuitry and dynamics of human transcription factor regulatory networks. Cell 150, 1274–86 (2012). 22959076 10.1016/j.cell.2012.04.040 PMC3679407

[b6] Tsankov A. M. *et al.* Transcription factor binding dynamics during human ES cell differentiation. Nature 518, 344–9 (2015). 25693565 10.1038/nature14233 PMC4499331

[b7] Yosef N. *et al.* Dynamic regulatory network controlling TH17 cell differentiation. Nature 496, 461–8 (2013). 23467089 10.1038/nature11981 PMC3637864

[b8] Stergachis A. B. *et al.* Conservation of trans-acting circuitry during mammalian regulatory evolution. Nature 515, 365–70 (2014). 25409825 10.1038/nature13972 PMC4405208

[b9] Jiang Y. X., Ma Y. & Cheng Y. Transcriptome and coexpression network analysis of the human glioma cell line Hs683 exposed to candoxin. J Int Med Res 40, 887–98 (2012). 22906261 10.1177/147323001204000307

[b10] Weller M. *et al.* Molecular predictors of progression-free and overall survival in patients with newly diagnosed glioblastoma: a prospective translational study of the German Glioma Network. J Clin Oncol 27, 5743–50 (2009). 19805672 10.1200/JCO.2009.23.0805

[b11] Ivliev A. E., t Hoen P. A. & Sergeeva M. G. Coexpression network analysis identifies transcriptional modules related to proastrocytic differentiation and sprouty signaling in glioma. Cancer Res 70, 10060–70 (2010). 21159630 10.1158/0008-5472.CAN-10-2465

[b12] Wei B. *et al.* Identification of differentially expressed genes regulated by transcription factors in glioblastomas by bioinformatics analysis. Mol Med Rep 11, 2548–54 (2015). 25514975 10.3892/mmr.2014.3094 PMC4337481

[b13] Baritaki S. *et al.* YY1 Over-expression in human brain gliomas and meningiomas correlates with TGF-beta1, IGF-1 and FGF-2 mRNA levels. Cancer Invest 27, 184–92 (2009). 19235591 10.1080/07357900802210760

[b14] Bhatia B., Hsieh M., Kenney A. M. & Nahle Z. Mitogenic Sonic hedgehog signaling drives E2F1-dependent lipogenesis in progenitor cells and medulloblastoma. Oncogene 30, 410–22 (2011). 20890301 10.1038/onc.2010.454 PMC3072890

[b15] Takahashi Y., Rayman J. B. & Dynlacht B. D. Analysis of promoter binding by the E2F and pRB families *in vivo*: distinct E2F proteins mediate activation and repression. Genes Dev 14, 804–16 (2000). 10766737 PMC316494

[b16] Xiao Y. *et al.* Identifying dysfunctional miRNA-mRNA regulatory modules by inverse activation, cofunction, and high interconnection of target genes: a case study of glioblastoma. Neuro Oncol 15, 818–28 (2013). 23516263 10.1093/neuonc/not018 PMC3688007

[b17] Xu J. *et al.* MiRNA-miRNA synergistic network: construction via co-regulating functional modules and disease miRNA topological features. Nucleic Acids Res 39, 825–36 (2011). 20929877 10.1093/nar/gkq832 PMC3035454

[b18] Li Y. *et al.* Comprehensive analysis of the functional microRNA-mRNA regulatory network identifies miRNA signatures associated with glioma malignant progression. Nucleic Acids Res 41, e203 (2013). 24194606 10.1093/nar/gkt1054 PMC3905890

[b19] Hu Y. *et al.* Identifying novel glioma associated pathways based on systems biology level meta-analysis. BMC Syst Biol 7 Suppl 2, S9 (2013). 10.1186/1752-0509-7-S2-S9 PMC3866263 24565222

[b20] Bruggeman F. J., Hornberg J. J., Boogerd F. C. & Westerhoff H. V. Introduction to systems biology. EXS 97, 1–19 (2007). 17432261 10.1007/978-3-7643-7439-6_1

[b21] Califano A. Rewiring makes the difference. Mol Syst Biol 7, 463 (2011). 21245848 10.1038/msb.2010.117 PMC3049406

[b22] Carro M. S. *et al.* The transcriptional network for mesenchymal transformation of brain tumours. Nature 463, 318–25 (2010). 20032975 10.1038/nature08712 PMC4011561

[b23] Matys V. *et al.* TRANSFAC: transcriptional regulation, from patterns to profiles. Nucleic Acids Res 31, 374–8 (2003). 12520026 10.1093/nar/gkg108 PMC165555

[b24] Milo R. *et al.* Network motifs: simple building blocks of complex networks. Science 298, 824–7 (2002). 12399590 10.1126/science.298.5594.824

[b25] Yu H. & Gerstein M. Genomic analysis of the hierarchical structure of regulatory networks. Proc Natl Acad Sci USA 103, 14724–31 (2006). 17003135 10.1073/pnas.0508637103 PMC1595419

[b26] Cheng C. *et al.* Construction and analysis of an integrated regulatory network derived from high-throughput sequencing data. PLoS Comput Biol 7, e1002190 (2011). 22125477 10.1371/journal.pcbi.1002190 PMC3219617

[b27] Jothi R. *et al.* Genomic analysis reveals a tight link between transcription factor dynamics and regulatory network architecture. Mol Syst Biol 5, 294 (2009). 19690563 10.1038/msb.2009.52 PMC2736650

[b28] Boyle A. P. *et al.* Comparative analysis of regulatory information and circuits across distant species. Nature 512, 453–6 (2014). 25164757 10.1038/nature13668 PMC4336544

[b29] Barresi V. *et al.* p-CREB expression in human gliomas: potential use in the differential diagnosis between astrocytoma and oligodendroglioma. Hum Pathol 46, 231–8 (2015). 25476123 10.1016/j.humpath.2014.10.011

[b30] Tan X. *et al.* cAMP response element-binding protein promotes gliomagenesis by modulating the expression of oncogenic microRNA-23a. Proc Natl Acad Sci USA 109, 15805–10 (2012). 23019365 10.1073/pnas.1207787109 PMC3465427

[b31] Murugan R. Theory on the dynamics of feedforward loops in the transcription factor networks. PLoS One 7, e41027 (2012). 22911735 10.1371/journal.pone.0041027 PMC3401222

[b32] Perry C. *et al.* Complex regulation of acetylcholinesterase gene expression in human brain tumors. Oncogene 21, 8428–41 (2002). 12466963 10.1038/sj.onc.1205945

[b33] Huang G. L. *et al.* Oncogenic activity of retinoic acid receptor gamma is exhibited through activation of the Akt/NF-kappaB and Wnt/beta-catenin pathways in cholangiocarcinoma. Mol Cell Biol 33, 3416–25 (2013). 23798555 10.1128/MCB.00384-13 PMC3753848

[b34] Liu R., Guo C. X. & Zhou H. H. Network-based approach to identify prognostic biomarkers for estrogen receptor-positive breast cancer treatment with tamoxifen. Cancer Biol Ther 16, 317–24 (2015). 25756514 10.1080/15384047.2014.1002360 PMC4622923

[b35] Yang Y. *et al.* Gene co-expression network analysis reveals common system-level properties of prognostic genes across cancer types. Nat Commun 5, 3231 (2014). 24488081 10.1038/ncomms4231 PMC3951205

[b36] Yang J. H., Li J. H., Jiang S., Zhou H. & Qu L. H. ChIPBase: a database for decoding the transcriptional regulation of long non-coding RNA and microRNA genes from ChIP-Seq data. Nucleic Acids Res 41, D177–87 (2013). 23161675 10.1093/nar/gks1060 PMC3531181

[b37] Gentleman R. C. *et al.* Bioconductor: open software development for computational biology and bioinformatics. Genome Biol 5, R80 (2004). 15461798 10.1186/gb-2004-5-10-r80 PMC545600

[b38] Irizarry R. A. *et al.* Summaries of Affymetrix GeneChip probe level data. Nucleic Acids Res 31, e15 (2003). 12582260 10.1093/nar/gng015 PMC150247

[b39] Wingender E., Dietze P., Karas H. & Knuppel R. TRANSFAC: a database on transcription factors and their DNA binding sites. Nucleic Acids Res 24, 238–41 (1996). 8594589 10.1093/nar/24.1.238 PMC145586

[b40] Kent W. J. *et al.* The human genome browser at UCSC. Genome Res 12, 996–1006 (2002). 12045153 10.1101/gr.229102 PMC186604

[b41] Sun J., Gong X., Purow B. & Zhao Z. Uncovering MicroRNA and transcription factor mediated regulatory networks in glioblastoma. PLoS Comput Biol 8, e1002488 (2012). 22829753 10.1371/journal.pcbi.1002488 PMC3400583

[b42] Krzywinski M. *et al.* Circos: an information aesthetic for comparative genomics. Genome Res 19, 1639–45 (2009). 19541911 10.1101/gr.092759.109 PMC2752132

[b43] Yu S. L. *et al.* MicroRNA signature predicts survival and relapse in lung cancer. Cancer Cell 13, 48–57 (2008). 18167339 10.1016/j.ccr.2007.12.008

[b44] Simon R., Radmacher M. D., Dobbin K. & McShane L. M. Pitfalls in the use of DNA microarray data for diagnostic and prognostic classification. J Natl Cancer Inst 95, 14–8 (2003). 12509396 10.1093/jnci/95.1.14

