# Supplementary material for: Construction and analysis of dynamic transcription factor regulatory networks in the progression of glioma
Source: Sci Rep. 2015 Nov 3;5:15953. doi: 10.1038/srep15953 (PMC4630656; doi:10.1038/srep15953)
Supplement: Supplementary Information [file srep15953-s1.pdf]

Construction and analysis of dynamic transcription factor regulatory networks in the progression of glioma

Yongsheng Li[1,2], Tingting Shao[1,2], Chunjie Jiang[1,2], Jing Bai[1], Zishan Wang[1], Jinwen Zhang[1], Lili Zhang[1], Zheng Zhao[1], Juan Xu[1,*], Xia Li[1,*]

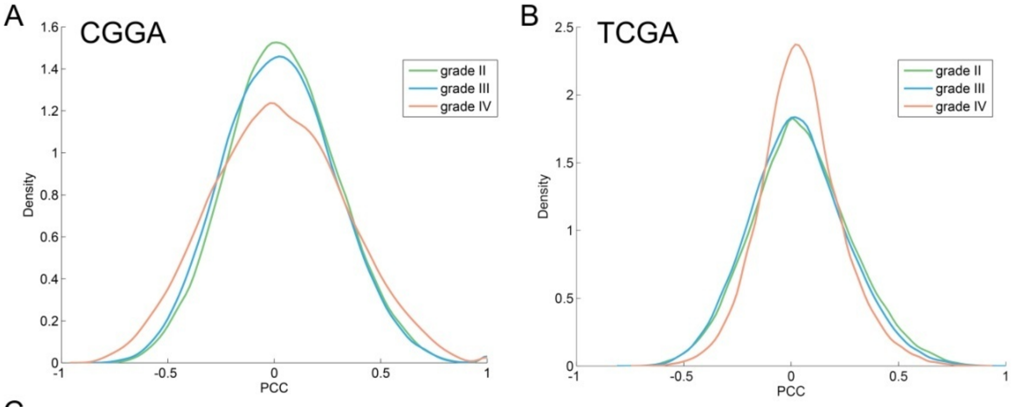

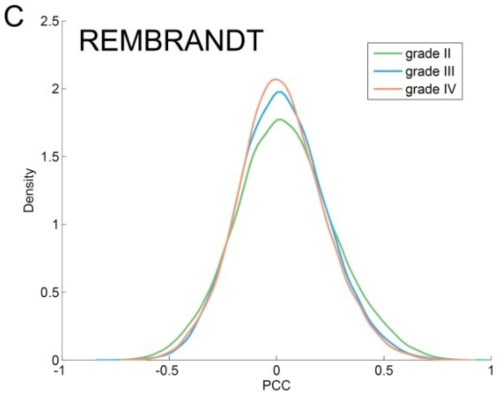

D

The KS test of the distribution of the correlation coefficient among TFs.

datasets	II VS III	II VS IV	III VS IV
CGGA	2.26E-37	2.15E-214	9.55E-105
TCGA	6.16E-37	8.06E-151	8.46E-211
REMBRANDT	2.79E-57	2.23E-118	1.36E-21

Figure S1. The distribution of correlation coefficient of TF-TF pairs were significantly distinct in three subtypes. A, CGGA dataset. B, TCGA dataset. C, REMBRANDT dataset. D, the KS test for the distribution of the corelation coefficient among TFs in three grades.

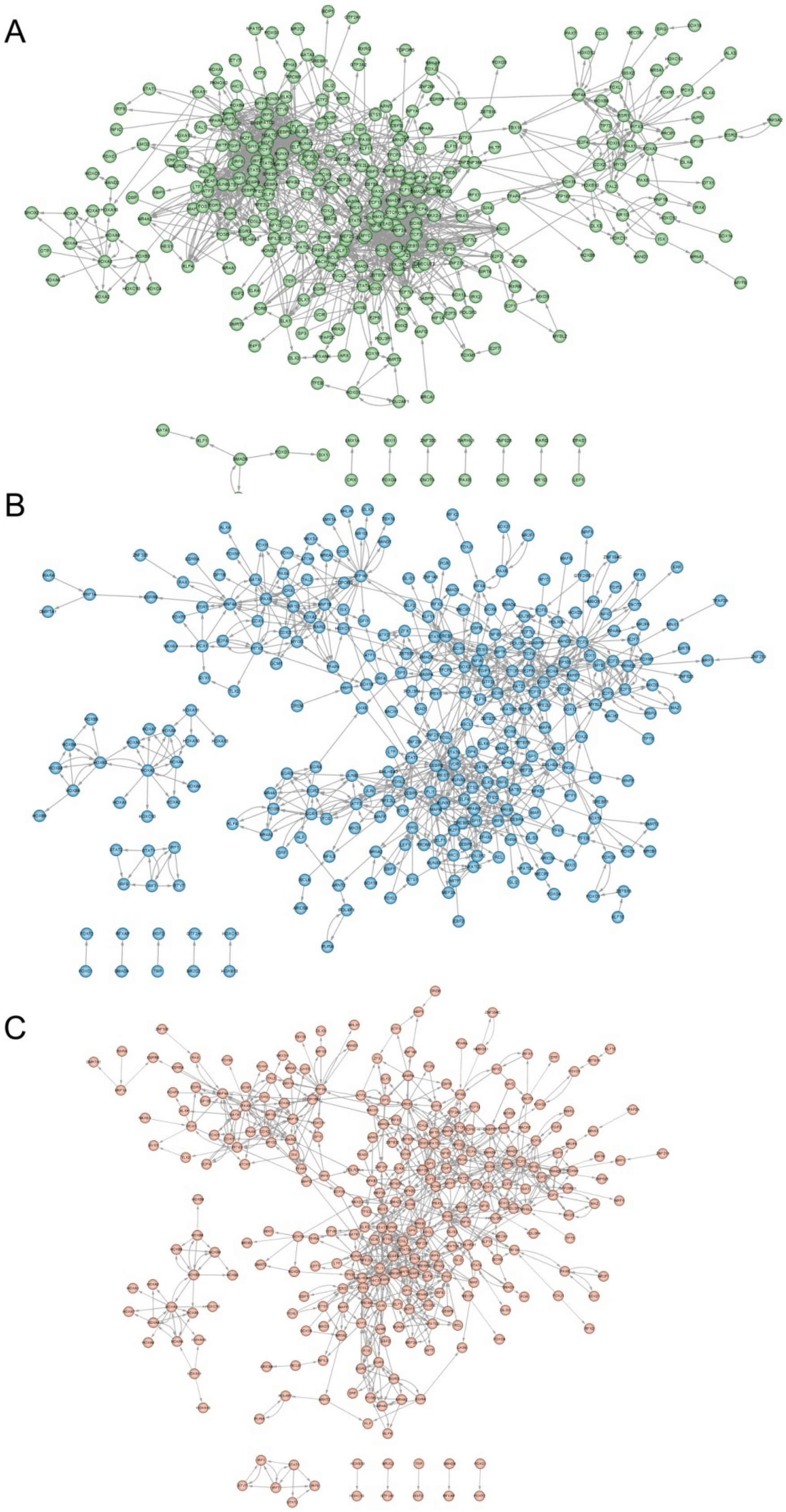

Figure S2. The transcription regulatory networks in three grades. A, grade II. B, grade III. C, grade IV.

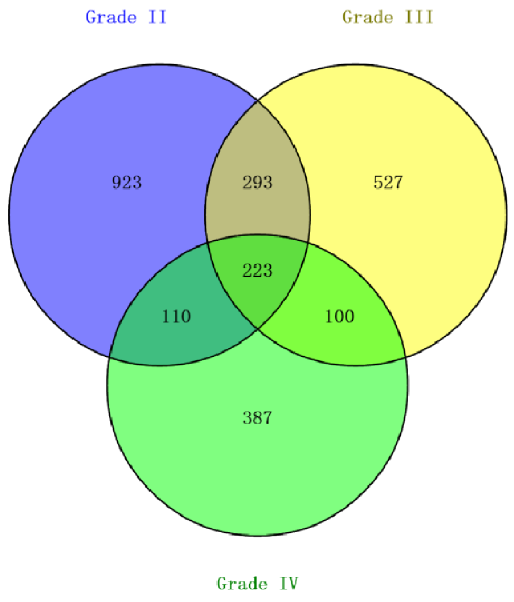

Figure S3. The venny plot shows that most TF-TF interactions are unique to specific grade.

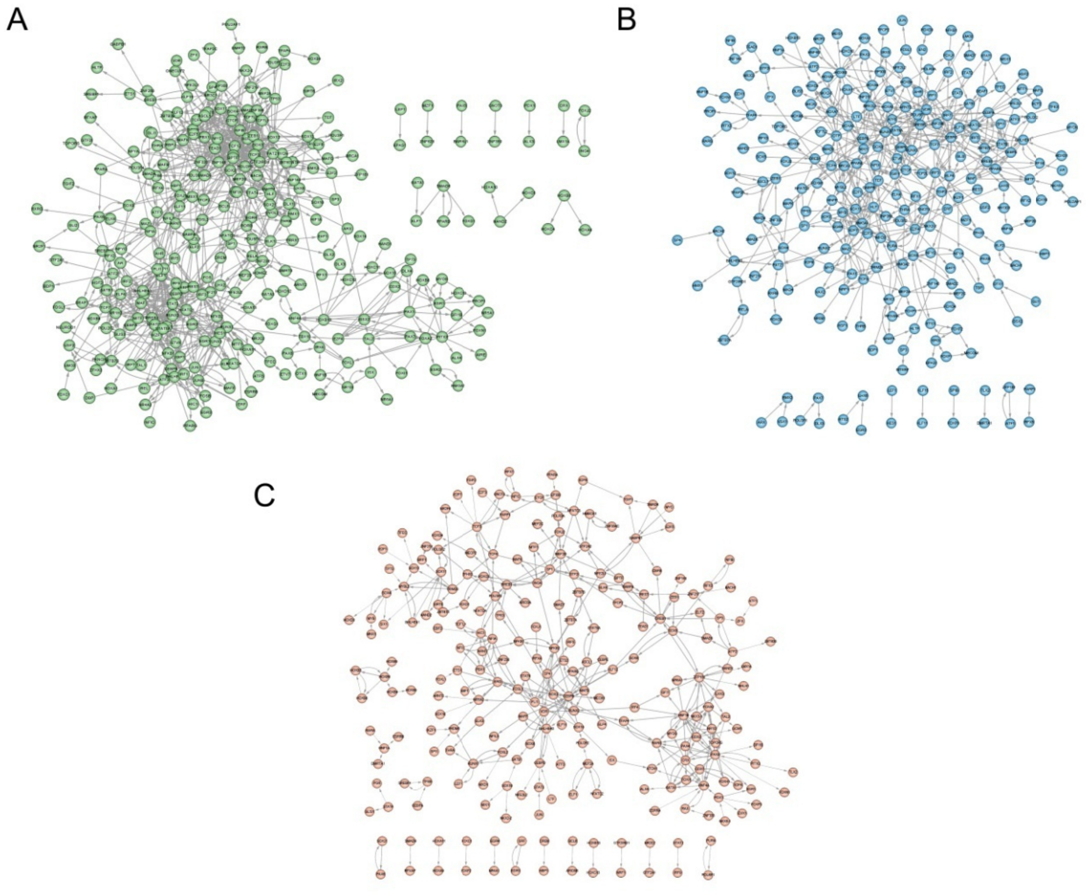

Figure S4. Cytoscape derived network showing all edges that are grade-specific in glioma.

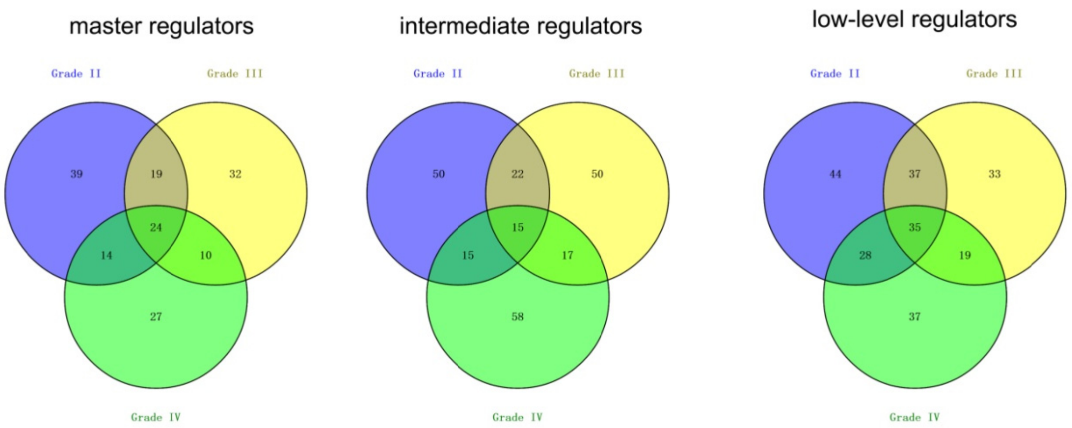

Figure S5. The venny plots show the overlap of master regulators, intermediate regulators and low-level regulators in three grades of glioma. The overlaps between distant grades are lower than that of adjacent grades.

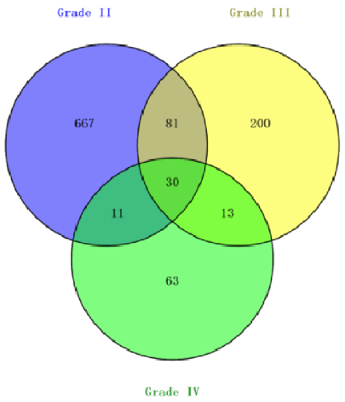

Figure S6. The overlap of FFLs in three grades of glioma.

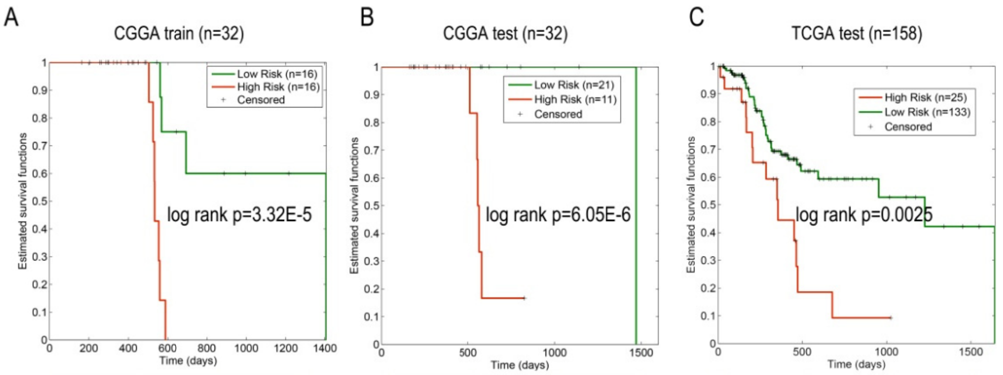

	IDH mut	IDH wild
Low Risk	2	12
High Risk	4	9

Fisher's exact test P=0.385

	IDH mut	IDH wild
Low Risk	1	18
High Risk	3	5

Fisher's exact test P=0.07

	IDH mut	IDH wild
Low Risk	3	25
High Risk	5	133

Fisher's exact test P=0.134

Figure S7. Network motifs are associated with the survival of grade IV patients. (A) Kaplan-Meier survival plots were generated for this motif using the CGGA train dataset. (B) Kaplan-Meier survival plots were generated for this motif using the CGGA test dataset. (C) Kaplan-Meier survival plots were generated for this motif using the TCGA dataset.

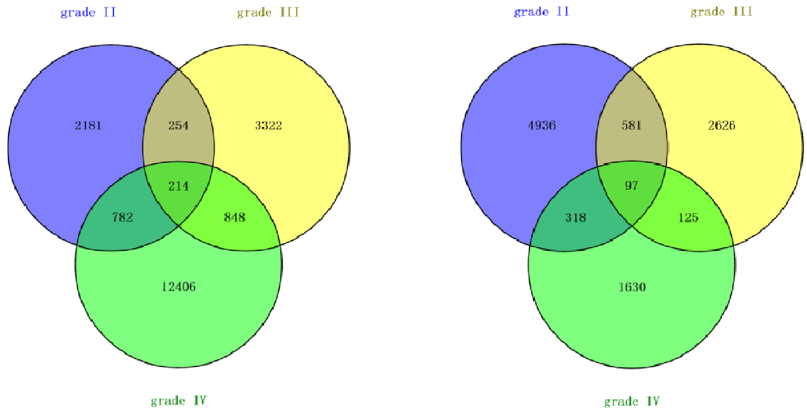

Figure S8. The overlap of grade specific regulatory networks by considering IDH mutation states. The first venny for IDH wild type and the second venny for IDH mutation type.

Table S1. The proportion of TF-TF regulations obtained by using IDH wild and mutated samples.

Grade	IDH wild	IDH mutated	Union
II	25.60%	70.92%	79.78%
III	50.43%	29.63%	69.19%
IV	96.39%	5.68%	96.83%

Table S2. The clinical information of the CGGA samples (Table S2.xls).

Table S3. Clinicopathologic characteristics of patients with GBM in the TCGA cohort (n = 158).

Characteristics	Number of patients		P
	**Training set** N=79	**Test set** N=79	
Sex			0.11 [a]
Female	49	53	
Male	30	26	
Age			0.64 [b]
Mean±SD	59.29±13.17	60.26±13.43	
Range	21-85	30-89	
IDH1 mutation			0.36 [a]
Mutated	5	3	
Wild	74	76	
Survival (day)			0.18 [b]
Mean±SD	385.92±326.16	322.60+268.60	
Range	6-1642	5-1448	
State			0.99 [a]
Living	26	26	
Death	53	53	

Abbreviations: IDH1, isocitrate dehydrogenase 1; SD, standard deviation.

[a] P values were determined using Fisher's exact test.

[b] P values were determined using student t test.
